# Molecular Dynamics Simulation Studies on the Aggregation of Amyloid-β Peptides and Their Disaggregation by Ultrasonic Wave and Infrared Laser Irradiation

**DOI:** 10.3390/molecules27082483

**Published:** 2022-04-12

**Authors:** Hisashi Okumura, Satoru G. Itoh

**Affiliations:** 1 Exploratory Research Center on Life and Living Systems, National Institutes of Natural Sciences, Okazaki 444-8787, Aichi, Japan; itoh@ims.ac.jp; 2 Institute for Molecular Science, National Institutes of Natural Sciences, Okazaki 444-8787, Aichi, Japan; 3 Department of Structural Molecular Science, SOKENDAI (The Graduate University for Advanced Studies), Okazaki 444-8787, Aichi, Japan

**Keywords:** molecular dynamics simulation, replica permutation method, amyloid-β, aggregation, disaggregation, β-sheet, α-helix, interface, inhibitor, polyphenol

## Abstract

Alzheimer’s disease is understood to be caused by amyloid fibrils and oligomers formed by aggregated amyloid-β (Aβ) peptides. This review article presents molecular dynamics (MD) simulation studies of Aβ peptides and Aβ fragments on their aggregation, aggregation inhibition, amyloid fibril conformations in equilibrium, and disruption of the amyloid fibril by ultrasonic wave and infrared laser irradiation. In the aggregation of Aβ, a β-hairpin structure promotes the formation of intermolecular β-sheet structures. Aβ peptides tend to exist at hydrophilic/hydrophobic interfaces and form more β-hairpin structures than in bulk water. These facts are the reasons why the aggregation is accelerated at the interface. We also explain how polyphenols, which are attracting attention as aggregation inhibitors of Aβ peptides, interact with Aβ. An MD simulation study of the Aβ amyloid fibrils in equilibrium is also presented: the Aβ amyloid fibril has a different structure at one end from that at the other end. The amyloid fibrils can be destroyed by ultrasonic wave and infrared laser irradiation. The molecular mechanisms of these amyloid fibril disruptions are also explained, particularly focusing on the function of water molecules. Finally, we discuss the prospects for developing treatments for Alzheimer’s disease using MD simulations.

## 1. Introduction

Proteins are normally folded correctly in vivo to maintain their functions. However, when their concentration increases due to, for example, aging, they aggregate to form oligomers, spherical aggregates, and amyloid fibrils, needle-like aggregates. These protein aggregates are associated with about 40 human neurodegenerative diseases [1,2,3]. For instance, amyloid-β (Aβ) peptide is related to Alzheimer’s disease. Huntington’s disease is caused by polyglutamine. Parkinson’s disease is associated with α-synuclein. Dialysis-related amyloidosis is caused by β2-microglobulin.

Alzheimer’s disease is one of dementia and is characterized by brain atrophy and senile plaques in the cerebral cortex [4,5]. The senile plaques are caused by the deposition of Aβ peptides on the brain cells [6,7]. Aβ is produced by proteolytic cleavage of the amyloid precursor protein and consists of 39–43 amino acid residues [8]. It usually consists of 40 or 42 amino acid residues. Aβ peptide with 40 residues is referred to as Aβ40, and that with 42 residues is referred to as Aβ42. The amino acid sequence of Aβ40 is DAEFRHDSGYEVHHQKLVFFAEDVGSNKGAIIGLMVGGVV, and that of Aβ42 is DAEFRHDSGYEVHHQKLVFFAEDVGSNKGAIIGLMVGGVVIA.

The structure of the Aβ amyloid fibril has been revealed by several experiments [9,10,11,12,13,14]. The main secondary structure of the Aβ amyloid fibril is the cross-β-sheet structure [9]. Aβ peptides form two intermolecular β-sheet structures, β1 and β2 [10,11]. The β1 and β2 regions consist of residues 12–24 and residues 30–40, respectively, in Aβ40 [10], while the β1 and β2 regions consist of residues 18–26 and residues 31–42, respectively, in Aβ42 [11]. The structures of individual Aβ peptides in the amyloid fibril models reported in Refs. [10,11] seem to be U-shaped. Other structural models have been also reported because Aβ peptides form polymorphic amyloid fibrils with various molecular structures depending on experimental conditions. For example, Lu et al. reported a three-fold symmetric amyloid fibril model consisting of three Aβ40 peptides [12]. The structure of Aβ42 in an amyloid fibril revealed by Xiao et al. is S-shaped [13]. Gremer et al. reported that the N-terminus of Aβ42 is L-shaped, and the C-terminus is S-shaped, giving the overall Aβ42 peptide an LS-shaped structure in their amyloid fibril model [14].

**Figure 1:**
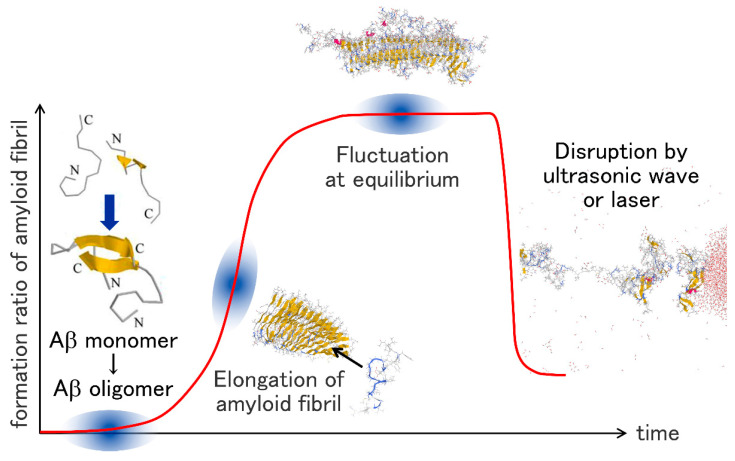
Schematic illustration of oligomerization of Aβ peptides, elongation of the Aβ amyloid fibril, the Aβ amyloid fibril in equilibrium, and disruption of the Aβ amyloid fibril.

The typical time course of the amyloid fibril formation is shown in Figure 1. First, several Aβ monomers aggregate to form an oligomer. The oligomer then grows to an amyloid fibril. Aβ peptides are attached to the ends of the amyloid fibril, making the amyloid fibril elongate. When almost all Aβ peptides in the solution aggregate, the system reaches thermal equilibrium, and the amyloid fibril stops the elongation. The amyloid fibril can be destroyed by ultrasonic wave irradiation or infrared laser irradiation.

The structural changes in the aggregation and disaggregation process have been investigated by molecular dynamics (MD) simulation. Numerous simulation studies have been performed so far on the monomeric state [15,16,17,18,19,20,21,22,23,24,25,26,27,28,29,30,31,32], dimerization [33,34,35,36,37,38,39,40,41,42,43,44,45,46], oligomerization [47,48,49,50,51,52,53,54], amyloid fibril elongation [55,56,57,58,59,60,61,62,63,64,65,66,67,68], amyloid fibril stability [69,70,71,72,73,74,75,76,77,78,79], and destruction of amyloid fibrils [80,81,82,83,84]. Most of these studies are well summarized in the review articles [85,86,87,88]. In this review, we explain the MD simulation studies on the aggregation and disaggregation of Aβ peptides that we have performed. These studies have elucidated the process from aggregation to disaggregation of the Aβ peptides at the atomic level. In Section 2, we present an MD simulation study on the aggregation process of Aβ fragments that revealed that the β-hairpin structure promotes the formation of the intermolecular β-sheet structure [36]. In Section 3, we explain that Aβ peptides at hydrophilic/hydrophobic interfaces form more β-hairpin structures than in the bulk water [30]. This is one of the reasons why aggregation at the interface is promoted. Research on the inhibition of aggregation of Aβ peptides has been ongoing, as well as their aggregation. Polyphenols have attracted attention as aggregation inhibitors for Aβ peptides. In Section 4, we introduce an MD simulation study on the interaction between an Aβ fragment and polyphenols [31]. When almost all the Aβ peptides form amyloid fibrils in an aqueous solution, the system reaches equilibrium. An MD simulation study has recently revealed that the structures of the two ends of the Aβ amyloid fibril are different in equilibrium [72]. We describe this simulation study in Section 5. Amyloid fibrils can be destroyed by ultrasonic wave irradiation or infrared laser irradiation. In Section 6, we explain an MD simulation study revealing that the cavitation induced by the ultrasonic wave destroys the amyloid fibrils [80]. In Section 7, we introduce an MD simulation study that clarified the function of water molecules in laser-induced amyloid fibril destruction [84]. Section 8 is devoted to the conclusions.

## 2. Aggregation of Aβ Fragments

To identify the important regions and amino acids in the amyloid fibril and oligomer formation of Aβ peptides, several experiments have been performed using the full-length Aβ peptides and Aβ fragments [89,90,91,92,93]. These studies revealed that the C-terminal region of the Aβ peptide, Aβ(29–42), consisting of the 29th to 42nd amino acid residues, promotes the amyloid fibril formation of the Aβ peptides [89]. Aβ(29–42) was also known to form amyloid fibrils by itself [90,91,92]. In the early stages of amyloid fibril formation, oligomers are formed. Recent studies have shown that oligomers are more neurotoxic than amyloid fibrils [94,95]. To develop a remedy for Alzheimer’s disease, it is necessary to understand the details of the oligomer structure and formation process of the Aβ peptides, but these are not clear. We recently investigated the oligomer formation process of the Aβ(29–42) peptides by MD simulation [36,50,96]. We introduce in this section the MD simulation study on the Aβ(29–42) dimerization [36].

### 2.1. Hamiltonian Replica-Permutation Molecular Dynamics Simulation of Aβ(29–42) Peptides

**Figure 2:**
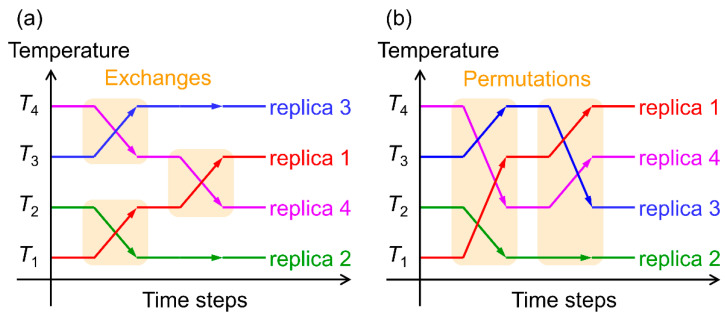
(**a**) Schematic illustration of time series of temperatures in the replica-exchange method. Orange squares mean replica exchange trials with the Metroplis algorithm. (**b**) Schematic illustration of time series of temperatures in the replica-permutation method. Orange rectangles mean replica permutation trials with the Suwa–Todo algorithm.

We performed Hamiltonian replica-permutation MD simulations of two Aβ(29–42) peptides in explicit water solvent [36]. The replica-permutation method [97] is one of the generalized-ensemble algorithms [98,99,100,101] developed by the authors. This method is an improved alternative to the replica-exchange method [102,103]. In the replica-exchange and replica-permutation methods, several copies of the system, referred to as replicas, are prepared, and each replica is assigned a different temperature. The temperatures are exchanged between two replicas during the simulation in the replica-exchange method, as shown in Figure 2a. In the replica-permutation method, on the other hand, the temperatures are permuted between three or more replicas, as shown in Figure 2b. In addition, the Suwa–Todo algorithm [104] is used instead of the Metropolis algorithm [105] for the replica-permutation trials. The Suwa–Todo algorithm is the most efficient Monte Carlo method and is utilized in several generalized-ensemble algorithms [22,97,106,107,108,109,110]. The replica-permutation method is known to provide statistically more reliable data on biomolecular structures than the replica-exchange method [97,106].

There are several variations of the replica-permutation method [22,106,107,108], such as the Hamiltonian replica-permutation method [22], the isobaric-isothermal replica-permutation method [107], the replica sub-permutation method [106], and the replica-permutation with solute tempering [110]. In the Hamiltonian replica-permutation method, an artificial parameter is introduced in the potential energy, and each replica is assigned a different value for this parameter. Instead of the temperatures, the parameter values are permuted between three or more replicas during the MD simulations. The method used here is the Coulomb replica-permutation method [23], which is a kind of the Hamiltonian replica-permutation method [22]. In this method, a parameter is introduced in the electrostatic potential energy, and the values of this parameter are permuted.

The MD simulations were performed as follows. Two Aβ(29–42) molecules with explicit water molecules were first prepared in a cubic simulation box. The N-terminus and C-terminus of Aβ(29–42) were blocked by the acetyl group and the N-methyl group, respectively. The amino acid sequence was Ace-GAIIGLMVGGVVIA-Nme. The AMBER parm99SB force field [111] and TIP3P rigid-body model [112] were used for the Aβ(29–42) peptides and water molecules, respectively. Temperature was controlled at 300 K by the Nosé–Hoover thermostat [113,114,115]. Coulomb replica-permutation MD simulations were performed with eight replicas from three different initial conditions. The simulation time was 200 ns, including 10 ns equilibration, for each replica. The total time length of the production runs of the Coulomb replica-permutation MD simulations was 4.56 μs. Other simulation details can be found in Ref. [36].

### 2.2. Dimerization of Aβ(29–42) Peptides

**Figure 3:**
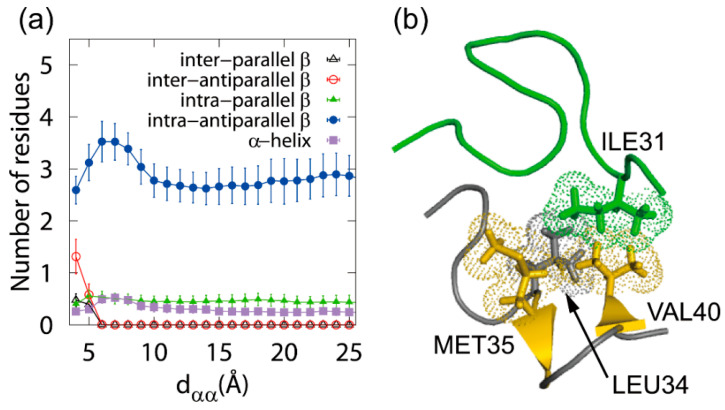
(**a**) The number of amino acid residues forming each secondary structure as a function of the intermolecular C_α_–C_α_ distance *d*_αα_ between the two Aβ(29–42) peptides. (**b**) A typical β-hairpin structure of Aβ(29–42). Reprinted with permission from Ref. [36]. Copyright 2014 American Chemical Society.

The dimerization of the Aβ(29–42) peptides was observed in the Coulomb replica-permutation MD simulations. The MD simulations showed that the dimer formation process proceeds in two steps. First, the β-hairpin structure increases when the two Aβ(29–42) molecules approach each other, as shown in Figure 3a, followed by the formation of a dimer with an intermolecular β-sheet structure. The reason for the increase in the β-hairpin structure in the first step is that a structure like Figure 3b becomes stable. In Figure 3b, Aβ(29–42) shown in yellow forms the β-hairpin structure, which is stabilized by the intermolecular hydrophobic side-chain contact between the amino acid residues shown by the yellow and green dots.

In the second step, it was found that the intermolecular β-sheet structures are readily formed at the amino acid residues with the intramolecular β-sheet structures. In other words, when the other Aβ(29–42) approaches the stable β-hairpin structure, the intermolecular β-sheet structure is easily formed between the β-hairpin and Aβ(29–42). In this way, the β-hairpin structure accelerates the formation of an oligomer with the intermolecular β-sheet structure. Not only our MD simulation study [36] but also some recent experimental and computational studies reported that the β-hairpin structure plays an essential role in the oligomer formation [35,116,117].

## 3. Structure of an Aβ Peptide at an Air–Water Interface

Aggregation of Aβ peptides is accelerated at hydrophilic/hydrophobic interfaces, such as air–water interfaces [118,119] and cell membrane surfaces [120,121]. One reason why the Aβ aggregation is accelerated there is that the concentration of Aβ peptides is higher at the interfaces because they have both hydrophobic and hydrophilic residues and tend to exist there. In addition, we recently performed MD simulations of Aβ40 at the air–water interface and found that it takes the β-hairpin structure more than in the bulk water [30]. As shown in the previous section, the β-hairpin structure promotes the intermolecular β-sheet formation. That is, the aggregation of Aβ peptides is enhanced not only by the high concentration but also by the conformation of the Aβ peptide. In this section, we explain the MD simulation study that revealed the structure of the full-length Aβ peptide, Aβ40, at the air–water interface [30].

### 3.1. Molecular Dynamics Simulation of Aβ40 at the Air–Water Interface

We performed MD simulations of an Aβ40 peptide in a system with air–water interfaces. The air–water interface was prepared by removing half the water molecules in a cubic simulation box. The side length of the box was set to 108.0 Å. For statistical analysis, nine different initial conditions were employed using the combination of three different coordinates and three different velocities. The initial structure of the Aβ40 peptide was fully extended with all dihedral angles *φ* and *ψ* of 180° for all the three initial coordinates. The MD simulation was performed from each initial condition for 240 ns including the equilibration period of 10 ns. Temperature was controlled at 350 K using the Nosé–Hoover thermostat [113,114,115].

For comparison, we also performed MD simulations of the Aβ40 peptide in the bulk water. The initial structure of the Aβ40 peptide in the bulk the water was also fully extended. Nine different initial conditions were prepared as well, with nine different initial velocities. The side length of the cubic unit cell was 91.1 Å. The MD simulation was performed from each initial condition for 240 ns including the equilibration period of 10 ns, again. For other simulation details, please refer to Ref. [30].

### 3.2. Molecular Structure of Aβ40 at the Air–Water Interface

**Figure 4:**
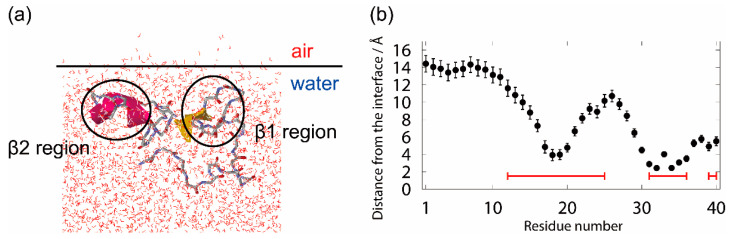
(**a**) A typical snapshot of the Aβ40 peptide at the interface. (**b**) The average distance between the C_α_ atoms of each amino acid residue of the Aβ40 peptide and the interface. The red lines represent the residues that were bound to the lyso-GM1 micelle in the experiment [122]. Reprinted with permission from Ref. [30]. Copyright 2019 American Chemical Society.

We observed that Aβ40 existed at the air–water interface in all MD simulations with the interface starting from nine different initial conditions. Figure 4a shows a typical conformation at the air–water interface. The β1 and β2 regions are bound at the interface, and the N-terminal region and the linker region between β1 and β2 are in the aqueous solution. These results mean that Aβ40 tends to exist at the air–water interface because the hydrophobic residues of Aβ40 tend to exist in the hydrophobic region (air), and the hydrophilic residues tend to exist in the hydrophilic region (water). That is, the Aβ peptide can be regarded as an amphiphilic molecule, such as a surfactant, and tends to exist at a hydrophilic/hydrophobic interface.

In order to clarify the Aβ40 structure at the interface, the average distance between the C_α_ atoms of each residue and the interface was calculated, as shown in Figure 4b. The positive value indicates that the C_α_ atom of that residue is in the water, and the negative value indicates that it is in the air. We can see that Aβ40 has an up-and-down shape at the air–water interface. This result agrees well with the NMR experiments for the Aβ40 structure on lyso-GM1 micelles [122], in which Val12–Gly25, Ile31–Val36, and Val39–Val40 of Aβ40 (red lines in Figure 4b) were found to bind to lyso-GM1 micelles. In addition, these results also agree with the Aβ40 conformation on GM1 micelles [123]. Thus, we can infer that the up-and-down shape of Aβ40 at the interface may hold for other hydrophilic/hydrophobic interfaces in general.

**Figure 5:**
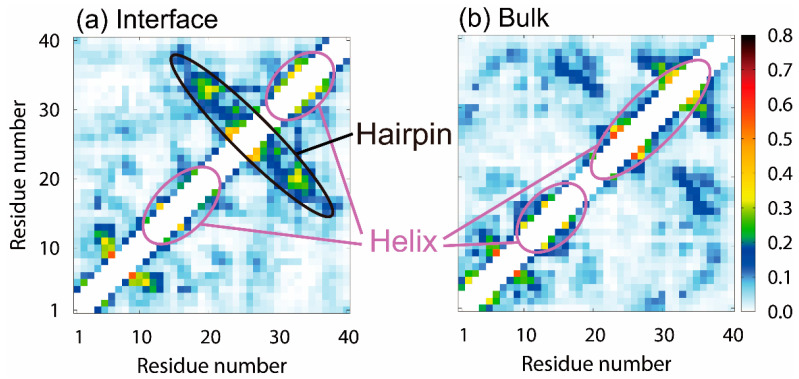
Intramolecular contact probabilities of the C_α_ atoms of Aβ40 (**a**) at the air–water interface and (**b**) in the bulk water. Reprinted with permission from Ref. [30]. Copyright 2019 American Chemical Society.

We calculated the intramolecular contact probabilities of the C_α_ atoms of Aβ40 to reveal the effect of the interface on the Aβ40 conformation. Figure 5a,b show the probabilities at the air–water interface and those in the bulk water, respectively. The β1 and β2 regions form helix structures at the air–water interface. This result agrees well with the experimental results on the lyso-GM1 micelles [122]. A β-hairpin structure is also formed between the β1 and β2 regions. These secondary structures were formed during the MD simulations as follows. The β1 and β2 regions first formed helix structures at the interface. The helix structure of the β1 region was then destroyed, and the extended β1 region approached the β2 region, forming a β-bridge. The helix structure in the β2 region was destroyed, and the β-hairpin structure was finally formed.

In the bulk water, on the other hand, helix structures are formed in the β1 and β2 regions, whereas the β-hairpin structure is hardly formed, as shown in Figure 5b. The difference between the β-hairpin formation probability at the interface and that in the bulk water causes a difference in the oligomer formation ability since the β-hairpin structure accelerates the intermolecular β-sheet formation with other Aβ peptides, as reviewed in the previous section [36,50]. This fact is also pointed out by experimental studies [116,117].

Thus, we can infer that there are two reasons why the aggregation of Aβ peptides is enhanced at the hydrophilic/hydrophobic interfaces. One reason is that the concentration of Aβ peptides increases at the interfaces since they have both hydrophilic and hydrophobic residues and tend to exist there. The other reason is that Aβ peptides take the β-hairpin structure, promoting aggregation.

**Figure 6:**
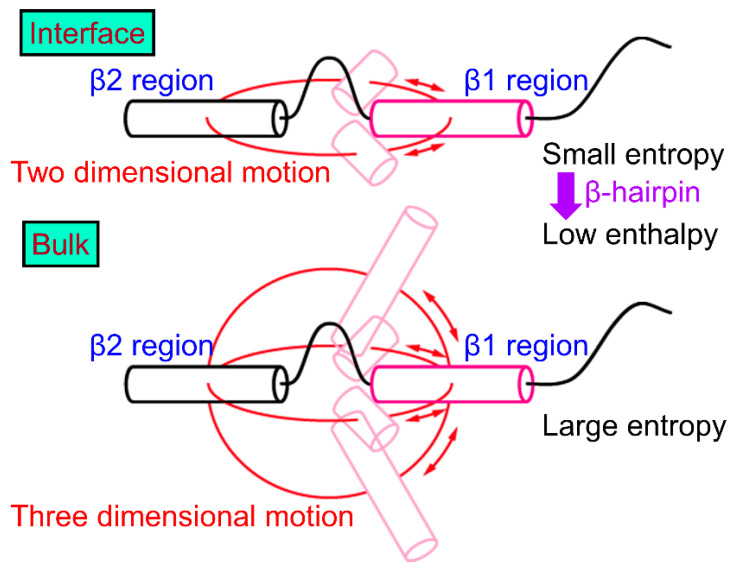
Schematic representation of the conformation of Aβ40 at the air–water interface and that in the bulk water. Reprinted with permission from Ref. [30]. Copyright 2019 American Chemical Society.

Next, we explain why the β-hairpin structure is stabilized at the hydrophilic/hydrophobic interface. Since the β1 and β2 regions tend to exist at the interface, as shown in Figure 4, these regions’ motion is restricted at the interface, that is, in two dimensions (Figure 6). In the bulk water, on the other hand, the β1 and β2 regions can move relatively freely in three dimensions. Entropy increases in the bulk water because the β1 and β2 regions can take more conformations. However, the entropy increase is suppressed due to the two-dimensional motion at the interface. To reduce the free energy, it is necessary to reduce enthalpy at the interface. Therefore, hydrogen bonds are formed between the β1 and β2 regions to reduce the enthalpy under this restriction. As a result, the β-hairpin structure is formed more at the interface.

We described here the structure of an Aβ peptide at the air–water interface. Several MD simulations have been performed to investigate the structure of an Aβ peptide at interfaces such as cell membrane surfaces, too [124,125,126,127,128,129,130,131]. An important membrane surface in the body is monosialotetrahexosylganglioside (GM1) clusters on neuronal cell membranes, because it is reported by experiments that Aβ peptide aggregation is promoted there [120,121]. MD simulation studies on the GM1 glycan cluster have also been performed [29,132]. The GM1 glycan cluster in these studies consists of a self-assembled supramolecule and GM1 glycans transplanted on it [133]. The HHQ region (residues 13–15) was found to bind well to the GM1 glycan cluster [29]. This fact is in good agreement with our results at the air–water interface, where the β1 region (residues 10–22) is present at the air–water interface. However, on the GM1 glycan cluster, Aβ formed an α-helix structure in the C-terminal region, but did not form the β-hairpin structure between the β1 and β2 regions. The reasons for this may be considered as follows. The GM1 glycan moiety on the self-assembled supramolecule has lower fluidity than the GM1 clusters on the neural cell membrane. Aβ, therefore, can reach only the GM1 glycan moiety that corresponds to the headgroup of the GM1 cluster on the membrane. The interface between the GM1 glycan region and the aqueous solution is not as different in hydrophilicity and hydrophobicity as the air–water interface because the GM1 glycan moiety is relatively hydrophilic. The reason for the β-hairpin formation is that the β1 and β2 regions are constrained at the hydrophilic/hydrophobic interface, as shown in Figure 6. Thus, we can consider that the β1 and β2 regions were not constrained on the GM1 glycan moieties of the GM1 glycan cluster as much as the air–water interface, and the β-hairpin structure was not formed on the GM1 glycan cluster. We expect that Aβ peptides can reach the interface between the GM1 glycan moiety and the lipid ceramide moiety and form the β-hairpin structure by performing MD simulations of Aβ with the GM1 clusters on the neural cell membrane in the future.

## 4. Inhibitor against Aggregation of Aβ Peptides: Polyphenol

Not only the aggregation of Aβ peptides but also the inhibition of the Aβ aggregation have been studied experimentally [134,135] and computationally [31]. It is known that the aggregation of Aβ peptides is inhibited by polyphenols [135]. The polyphenols thus have attracted attention as drug candidate molecules against Alzheimer’s disease. The efficiency in inhibiting the Aβ aggregation has been investigated for several polyphenols [135]. According to recent experiments, myricetin (Myr) and rosmarinic acid (RA) (Figure S1) are most effective in inhibiting the Aβ aggregation [135]. However, the molecular mechanism of these polyphenols inhibiting the Aβ aggregation is not revealed. We recently performed MD simulations of an Aβ(16–22) peptide and these polyphenols to gain insight into this problem [31]. The Aβ(16–22) peptides are known to form amyloid fibrils by experiments [93]. It is relatively easy to reproduce the intermolecular β-sheet formation by MD simulation [136,137,138,139,140]. We present the MD simulation study on the interaction between the Aβ(16–22) peptide and these polyphenols [31] in this section.

### 4.1. Replica-Permutation MD Simulation of an Aβ(16–22) Peptide and Polyphenols

We performed all-atom replica-permutation MD simulations of an Aβ(16–22) peptide and polyphenols [31]. Each system consists of one Aβ(16–22) peptide, one polyphenol molecule (Myr or RA), and water molecules. For the RA system, we added a Na^+^ ion as a counter ion. The N-terminus of the Aβ(16–22) peptide was blocked by the acetyl group, and the C-terminus by the N-methyl group to reduce the effect of the N- and C-terminal electric charges. The amino acid sequence is thus Ace-KLVFFAE-Nme. We used the AMBER parm14SB [141] and generalized AMBER force fields [142] for the Aβ(16–22) peptide and polyphenol molecules, respectively. The TIP3P rigid-body model [112] was used for the water molecules. To control the temperatures, the Nosé–Hoover thermostat [113,114,115] was used. We employed 14 replicas in the replica-permutation simulations. The temperatures of the replicas were ranged from 300.0 to 500.0 K. The Generalized-Ensemble Molecular Biophysics (GEMB) program was used to perform the MD simulations. This program was developed by one of the authors (H. Okumura) and has been applied to several protein and peptide systems [106,107,108,110,143,144,145,146,147,148,149,150,151,152,153,154,155]. We can perform MD simulations with the generalized-ensemble algorithms [98,99,100,156], such as the replica-exchange [102,103], replica-permutation [22,97,157], multicanonical [158,159,160,161], and multibaric-multithermal [162,163,164,165] methods, using this program. Here, a replica-permutation MD simulation was performed for 120 ns for each replica, including the first 20 ns as the equilibration. We then observed how these polyphenols bound to the Aβ(16–22) peptide. Other simulation details can be found in Ref. [31].

### 4.2. Structure of the Complexes of an Aβ(16–22) Peptide and Polyphenols

**Figure 7:**
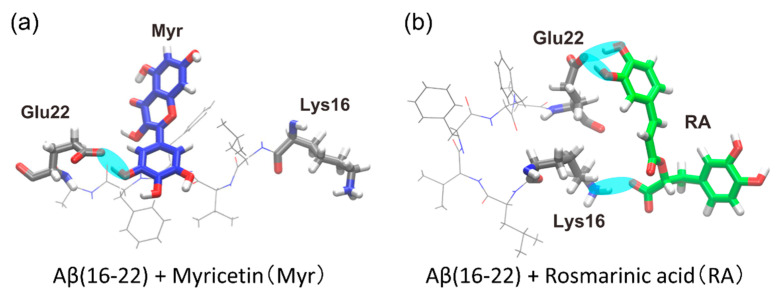
Typical snapshots obtained from the replica-permutation MD simulations of the (**a**) Myr system and (**b**) RA system. The hydrogen bonds between the polyphenols and the Aβ(16–22) peptide are indicated by the cyan ovals. Reprinted with permission from Ref. [31]. Copyright 2020 Elsevier.

As a result of the MD simulations, we observed that polyphenols were bound to the Aβ(16–22) peptide, as shown in Figure 7. Hydrogen bonds were formed, as indicated by the cyan ovals in Figure 7, between the polyphenols and Aβ(16–22) peptide. In the Myr system, the carboxyl group (-COO) of Glu22 often formed a hydrogen bond with a hydroxy group (-OH) of Myr, as shown in Figure 7a. In the RA system, the amine group (-NH3) of Lys16 often bound to the carboxyl group of RA, and the carboxyl group of Glu22 frequently formed a hydrogen bond with a hydroxy group of RA, as shown in Figure 7b.

**Figure 8:**
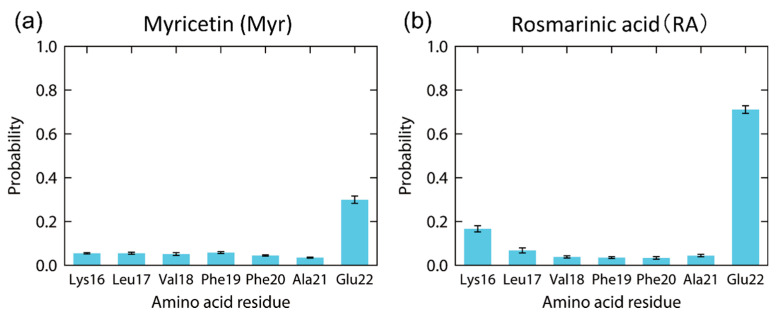
Contact probability of each residue in the Aβ(16–22) peptide with (**a**) Myr and (**b**) RA at 300 K. Reprinted with permission from Ref. [31]. Copyright 2020 Elsevier.

The contact probability of each amino acid residue of the Aβ(16–22) peptide with these polyphenols was also calculated, as in Figure 8. Myr binds to Glu22 with the probability of 30%, as shown in Figure 8a. However, the other residues of the Aβ(16–22) peptide have much lower contact probabilities with Myr. High contact probabilities in the RA system are found at two residues, Glu22 with 71% and Lys16 with 17%, as shown in Figure 8b. On the other hand, the hydrophobic residues (Leu, Val, Phe, and Ala) have low contact probabilities in both systems. It is known that the Aβ(16–22) peptides form anti-parallel β-sheets because of the electrostatic interaction between the carboxyl group of Glu22, which has a negative charge, and the amine group of Lys16, which has a positive charge [53,137]. We can thus expect that the aggregation of the Aβ(16–22) peptides is inhibited by Myr and RA because they bind to the side chains of Glu22 and Lys16, as shown in Figure 7.

**Figure 9:**
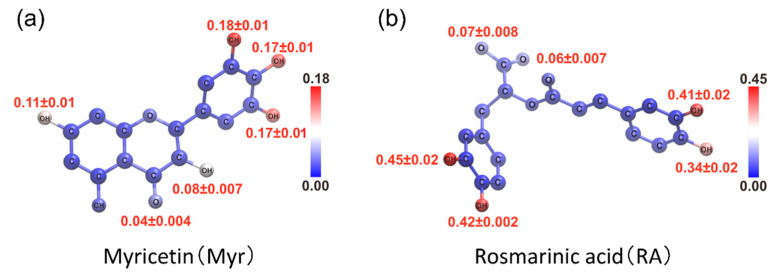
Color mapping to show contact probability of the (**a**) Myr and (**b**) RA atoms with the Aβ(16–22) peptide at 300 K. Reprinted with permission from Ref. [31]. Copyright 2020 Elsevier.

The contact probability of each atom of polyphenols was also calculated to specify which atoms of polyphenols contribute to the interaction with the Aβ(16–22) peptide, as shown in Figure 9. As a result, multiple adjacent hydroxy groups around six-membered rings were found to have high contact probabilities with the Aβ(16–22) peptide in both Myr and RA systems. The carboxyl group in RA also contacts the Aβ(16–22) peptide. Thus, we can expect that these atoms in polyphenols play an essential role in inhibiting the Aβ(16–22) aggregation.

## 5. Structures of the Two Ends of the Aβ Amyloid Fibril

**Figure 10:**
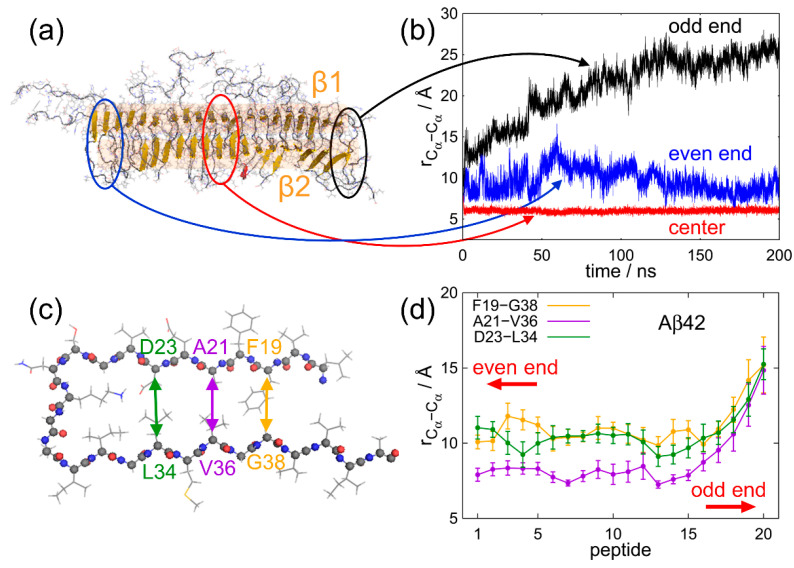
(**a**) A snapshot of the Aβ42 amyloid fibril in the MD simulation. (**b**) Time series of C_α_–C_α_ distance between A21 and V36 at the odd end, in the center region, and at the even end. (**c**) Side view of chain C of model 1 of the PDB conformation (PDB ID: 2BEG) of the Aβ42 amyloid fibril. (**d**) The average C_α_–C_α_ distances of the Aβ42 amyloid fibril between F19 and G38 (orange), A21 and V36 (purple), and D23 and L34 (green). Panels (**a**,**c**) were created using PyMOL [171]. Reprinted with permission from Ref. [72]. Copyright 2016 Springer Nature.

The structures of Aβ amyloid fibrils have been clarified by X-ray diffraction and solid-state NMR experiments [120,166,167]: the amyloid fibril has a cross-β structure comprising two β-sheets, β1 and β2, as shown in Figure 10a. Here, the β1 and β2 regions correspond to residues 18–26 and 31–42, respectively. However, it is generally known that the structure in the bulk region and that at the interface are different in many materials, known as the surface reconstruction of crystals [168] and polarization on water surface [169,170]. In the case of the amyloid fibril, the bulk region corresponds to the central part of the amyloid fibril, and the interface corresponds to the end of the amyloid fibril. The amyloid fibril structure revealed by the experiments is that in the central region. The structures at the ends of the amyloid fibril have not been revealed because only one or two Aβ peptides constitute the end of the amyloid fibril, which cannot be measured by experimental techniques such as X-rays and NMR. In addition, the amyloid fibril elongates by binding one Aβ peptide to the end of the fibril. It is thus important to clarify the structure of the Aβ peptide at the ends of the amyloid fibril to understand the elongation mechanism of the fibril.

We, therefore, performed MD simulations to investigate the structure of the amyloid fibril ends [72]. As a result, not only the difference in Aβ structure between the ends and the central region but also that between two ends were discovered. The two ends of the Aβ amyloid fibril are referred to as the odd and even ends because C=O and N–H of the odd-numbered (even-numbered) residues in the β1 region are exposed at the odd (even) end [11]. Different molecular conformations between the odd and even ends had not been reported before our MD simulations [10]. In this section, we introduce the MD simulation study to reveal the structural differences at the odd end, in the central region, and at the even end of the Aβ amyloid fibril [72].

### 5.1. Molecular Dynamics Simulation of the Aβ Amyloid Fibril

We prepared an amyloid fibril consisting of 20 Aβ42 peptides with explicit water molecules. Because the central structure of the Aβ amyloid fibril is known by solid-state NMR experiments (PDB: 2BEG) [11], the initial structures of the Aβ amyloid fibrils in the MD simulations were modeled using this structure. The AMBER parm99SB was used for the Aβ peptide force field [111], and the TIP3P rigid-body model was used for the water molecules [112]. The electrostatic interaction was calculated using the particle mesh Ewald method [172], and the time step width for the Aβ peptide was set to 0.5 fs and that for the water molecules to 4 fs. The water molecules were treated as rigid-body molecules [144]. The temperature was set to 298 K using the Nosé–Hoover thermostat [113,114,115], and the pressure was set to 0.1 MPa using the Andersen barostat [173]. Then, 200 ns simulations were performed from nine different initial conditions. We used the GEMB program [148] here again. For other simulation details, please refer to Ref. [72].

### 5.2. Structure of Aβ Peptides at the Ends of the Aβ Amyloid Fibril

**Figure 11:**
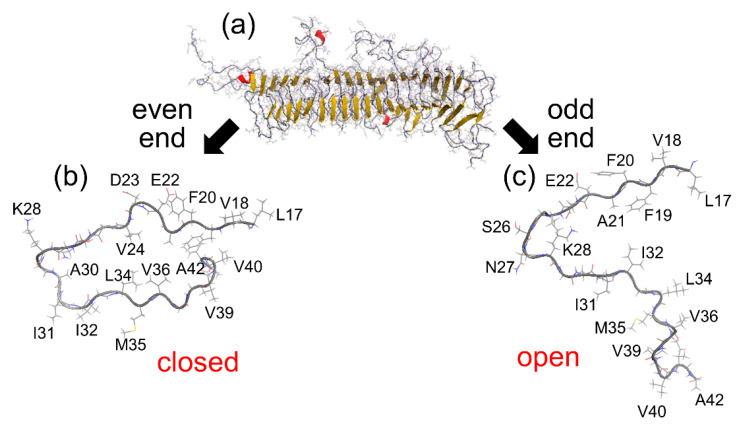
(**a**) A snapshot of the Aβ amyloid fibril. (**b**) Side view of the Aβ peptide at the even end. (**c**) Side view of the Aβ peptide at the odd end. The figures were created using PyMOL [171]. Reprinted with permission from Ref. [72]. Copyright 2016 Springer Nature.

We unexpectedly observed that the N- and C-termini gradually opened at the odd end, whereas these termini remained closed at the even end. In all simulations, the odd end often opened, whereas the even end never opened. Figure 10b shows the time series of the C_α_–C_α_ distance between A21 and V36 at the odd end, in the central region, and at the even end. The pair of C_α_ atoms of A21 and V36 is illustrated in Figure 10c. The C_α_–C_α_ distance between these residues clearly increased at the odd end. On the other hand, at the even end, this C_α_–C_α_ distance fluctuated, but did not increase so much. In the central region, it was almost constant. Figure 10d shows the averages of three C_α_–C_α_ distances between F19 and G38, A21 and V36, and D23 and L34. The averages were taken over the nine initial conditions at a time ranging from 100 to 200 ns. The differences in the three C_α_–C_α_ distances between the odd end and even ends are statistically significant. It means that not only from one MD trajectory but also after taking averages of nine MD trajectories, we can see that the β-sheets were well separated at the odd end, whereas the two β-sheets were closely spaced with some fluctuation at the even end. To illustrate this structural difference at both ends clearly, Figure 11 shows the Aβ amyloid fibril and the side views of the Aβ peptides at both ends.

**Figure 12:**
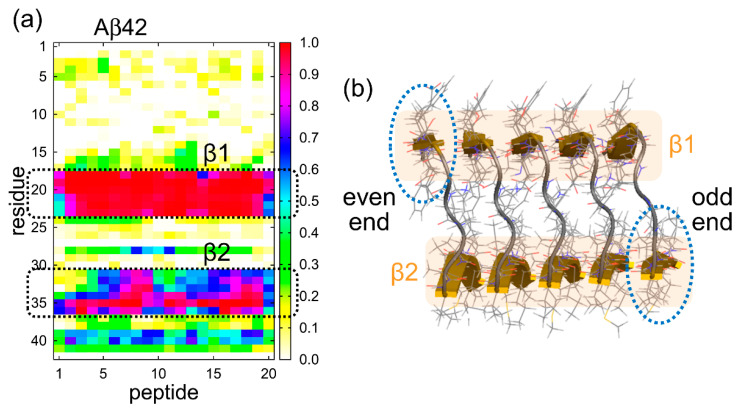
(**a**) The probability that each amino acid residue in each Aβ peptide has a parallel intermolecular β-sheet structure. (**b**) The Aβ amyloid fibril structure revealed by NMR experiments (PDB: 2BEG) [11]. The solvent-exposed β-strands at the even and odd ends are indicated by ellipses. Reprinted with permission from Ref. [72]. Copyright 2016 Springer Nature.

In order to explain why the structures and fluctuations differ between the two ends, we calculated the probability that each amino acid residue forms an intermolecular parallel β-sheet structure, as shown in Figure 12a. Since 20 Aβ42 peptides were used, the horizontal axis represents the peptide number (1–20) and the vertical axis represents the amino acid residue number (1–42). The β2 region has a high formation probability of the intermolecular parallel β-sheet structure, and the β1 region has a much higher probability than the β2 region. The reason for this difference is that β2 contains the glycine residues, which tend to move easily. This result explains the large fluctuation at the odd end as follows. We can see from the PDB structure that β2 does not exist directly below β1 because each Aβ peptide is slightly tilted, as shown in Figure 12b. Therefore, β1 is more exposed to the solvent at the even end, whereas β2 is more exposed to the solvent at the odd end, as indicated by the dashed ellipses in Figure 12b. These two β-strands, β1 at the even end and β2 at the odd end, are both exposed to the solvent and therefore tend to fluctuate. However, as shown in Figure 12a, β1 forms a more stable intermolecular β-sheet structure with the neighboring Aβ peptide, whereas β2 does not form such a stable intermolecular β-sheet structure with the neighboring Aβ peptide. Therefore, the odd end, where β2 is exposed, tends to fluctuate more and to take open and closed conformations.

It was experimentally known that the Aβ fibrils extend only in one direction [174,175]. This unidirectionality of the fibril extension implies that the odd and even ends take different conformations, but it was not clear what exactly the structures of both ends were. Our simulation study is the first work to reveal the difference in the structure and fluctuation between the two ends of an amyloid fibril.

After we performed the MD simulations, the structure of a single amyloid fibril of yeast prion protein sup35 was observed by high-speed atomic force microscopy [176]. This experiment showed that the fluctuation was large at one end and small at the other end, as we predicted from the MD simulations. In other words, the difference in the structures and fluctuations of the two ends of the amyloid fibril that we predicted was confirmed by the experiment.

## 6. Amyloid Fibril Disruption by Ultrasonic Waves

Amyloid fibrils can be destroyed by ultrasonic wave irradiation or infrared laser irradiation. It has been suggested that the destruction mechanism by the ultrasonic wave is due to cavitation (bubble formation), but the atomic-level details of how the bubbles in water destroy the amyloid fibrils have not been understood experimentally. MD simulation studies on cavitation had been performed mainly for simple liquids such as Lennard-Jones liquids [177,178,179,180], but not for biomolecular systems. We recently performed nonequilibrium MD simulations of the destruction of the Aβ amyloid fibril by applying ultrasonic waves [80]. In this section, we review the MD simulations of the amyloid fibril disruption by the ultrasonic waves.

### 6.1. Molecular Dynamics Simulation to Mimic Ultrasonic Waves

We prepared amyloid fibrils consisting of dodecamer, hexamer, and trimer of Aβ peptides with explicit water molecules. The numbers of water molecules are 10,168, 11,112, and 11,591 for the dodecamer, hexamer, and trimer systems, respectively. Twelve, six, and three sodium ions were also included as counter ions in the dodecamer, hexamer, and trimer systems, respectively. After equilibration MD simulations, nonequilibrium MD simulations were performed with time-dependent pressure to mimic the ultrasonic waves. This pressure is expressed by a sinusoidal curve, which is given by

(1) $$P(t)=P_{0}+\Delta P\sin\left(\frac{2\pi t}{\tau }\right),$$

where average pressure *P*_0_, pressure amplitude Δ*P*, and period *τ* were set as *P*_0_ = 100 MPa, Δ*P* = 200 MPa, and *τ* = 1 ns, as illustrated in Figure S2. The temperature was controlled at 298 K with the Nosé–Hoover thermostat [113,114,115]. The pressure was controlled with the Andersen barostat [173]. We used the AMBER parm99SB force field [111] for the Aβ peptides and the TIP3P rigid-body model [112] for the water molecules. The symplectic [181] quaternion scheme [144,182] was used for the water molecules. The same MD simulations were performed for 10 ns (=10*τ*) from 20 different initial conditions for statistical analysis. These MD simulations were performed with the GEMB program [148], again. For other simulation details, see Ref. [80].

### 6.2. Disruption of Aβ Amyloid Fibril by Ultrasonic Waves

**Figure 13:**
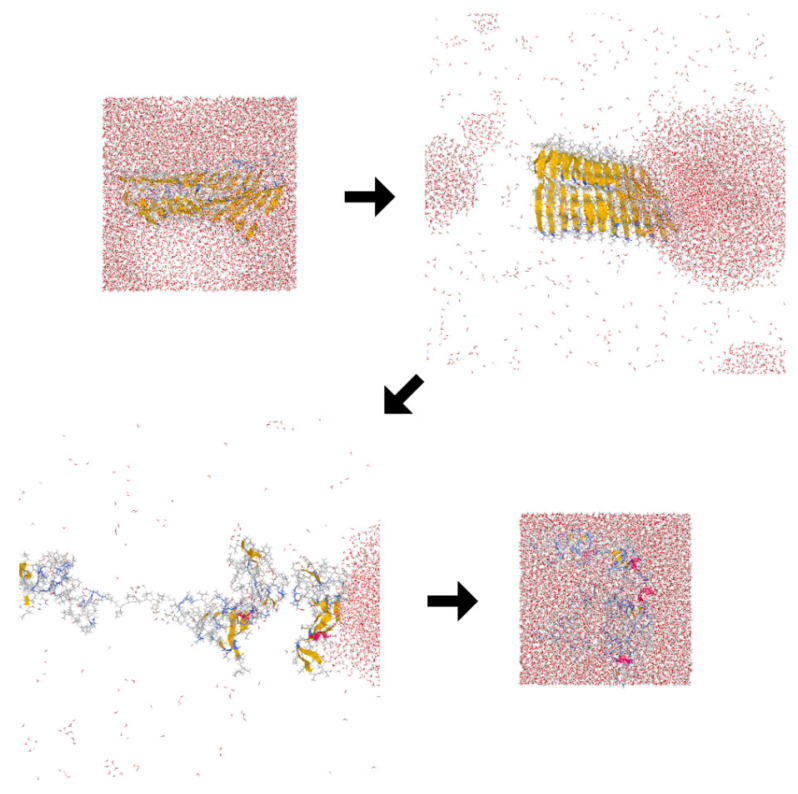
Snapshots of the destruction of the Aβ amyloid fibril by ultrasonic waves. The amyloid fibril was destroyed by the jet flow generated when the bubble collapsed. Reprinted with permission from Ref. [80]. Copyright 2014 American Chemical Society.

The disruption process of the Aβ amyloid fibril by the ultrasonic wave is shown in Figure 13. When the pressure was positive, there was no significant change in the amyloid fibril and water structure. However, when the pressure became negative, a bubble was generated around the amyloid fibril, often near the hydrophobic residues in the β2 region. When the pressure became positive again, the bubble collapsed and a water droplet attacked the amyloid fibrils as a jet flow, resulting in the disruption of the Aβ amyloid fibril.

**Figure 14:**
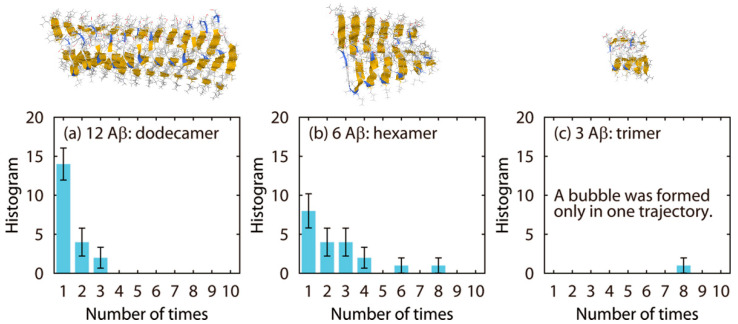
Histograms that show how many times the pressure had been negative before the amyloid fibril was destroyed for the (**a**) dodecamer, (**b**) hexamer, and (**c**) trimer systems. Reprinted with permission from Ref. [80]. Copyright 2014 American Chemical Society.

Once the amyloid fibril was destroyed, the bubble formation was not observed again. This result suggests that the hydrophobic residues in the β2 region serve as a nucleus for the bubble formation. Even if the same number of hydrophobic residues exist in the water, they cannot function as a nucleus unless assembled as the amyloid fibril. Therefore, we also performed nonequilibrium MD simulations of amyloid fibrils consisting of six and three Aβ peptides. Figure 14 shows how many times the pressure had been negative before the bubbles were formed and the amyloid fibrils were disrupted in twenty MD simulations for each system. In the dodecamer system, a bubble was formed at the first negative pressure in fourteen MD simulations. In four MD simulations, a bubble was formed at the second negative pressure. In two MD simulations, a bubble was formed at the third negative pressure. However, it takes longer for shorter amyloid fibrils to be destroyed. In the trimer system, in particular, a bubble was formed only in one MD simulation out of twenty simulations. These results mean that it takes longer for a shorter amyloid to be a nucleus for the bubble formation. Because the β2 region mainly consists of the hydrophobic residues, these residues can be the nucleus for the bubble formation. The hydrophobic residues in the short amyloid fibrils are not enough to function as a nucleus. This is why it takes time for the bubble formation in the case of short amyloid fibrils.

It was found in experiments that after amyloid fibrils were broken down into shorter fibrils by ultrasonication, the lengths of the short amyloid fibrils were almost the same [183]. This experimental result can be explained from our MD simulations as follows. If the amyloid fibril is longer than some critical length, the region with the hydrophobic residues can be large enough as the nucleus for the bubble formation, and the bubble breaks down the fibrils. On the other hand, if the amyloid fibril is not long enough, the hydrophobic region is not enough, and the amyloid fibrils are not disrupted. This is why ultrasonication makes the length of the amyloid fibril be almost the same.

## 7. Laser-Induced Disruption of the Aβ Amyloid Fibril

It is also known that amyloid fibrils can be broken down via infrared free-electron laser (IR-FEL) irradiation. The destruction of amyloid fibrils via laser irradiation has been studied using both experimental [184,185,186] and theoretical techniques [82]. Amyloid fibrils form intermolecular hydrogen bonds between backbone C=O and N–H. Therefore, it was assumed that when a laser that matches the frequency of the C=O stretching vibration is irradiated, the C=O stretching vibration resonates and is amplified, which breaks the hydrogen bonds and results in the disruption of the amyloid fibrils [82]. However, recent experiments showed that Aβ amyloid fibrils under dry conditions are not destroyed by the same laser irradiation; they are only destroyed in the presence of water [185]. This fact suggests that water molecules play an essential role in amyloid fibril destruction. However, the role of the water molecules had not been known.

As the last topic of this review, we introduce our recent MD simulations for the disruption of an Aβ amyloid fibril via laser irradiation in an aqueous solution [84]. In this study, we revealed a new role of water molecules in breaking hydrogen bonds in biomolecules; this mechanism is different from water penetration under high pressure [100,148,187,188,189] and water jets when ultrasonic waves are applied [80]. In addition, we succeeded in reproducing an experimental observation [185], in which more α-helix structures are formed after the laser irradiation, and explaining the reason for this phenomenon.

### 7.1. Molecular Dynamics Simulation to Mimic Laser Irradiation

In IR-FEL experiments, a sample is irradiated with an infrared laser that corresponds to the backbone C=O stretching vibration (amide I band). To determine the resonance wavenumber of the C=O stretching vibration of the model Aβ amyloid fibril, we first performed equilibrium MD simulations of an amyloid fibril consisting of twelve Aβ42 peptides in an explicit water solvent. We used the GEMB program [148] again to perform the MD simulations. The initial amyloid fibril conformations were prepared using model 1 of the 2BEG PDB conformation [11]. A total of 1 Aβ amyloid fibril, 36 sodium ions, and 25,480 water molecules were placed in a cubic simulation box with a side length of 96.324 Å. The total number of atoms was 84,000. Six different initial conditions were prepared for the statistical analysis.

We applied the AMBER parm14SB force field [141] to the Aβ peptides and counter ions. We used the TIP3P rigid-body model [112] for the water molecules by adopting the symplectic [181] quaternion scheme [144,182]. The MD simulations were performed at 310 K and 0.1 MPa for 50 ns from the six initial conditions. The temperature was controlled using the Nosé–Hoover thermostat [113,114,115]. The pressure was controlled using the Andersen barostat [173]. The first 10 ns of the simulations were regarded as the equilibration, and the following 40 ns were used for the analysis. We used the amino acid residues V18–D23 in the β1 region and I31–V36 in the β2 region to calculate the infrared absorption spectrum of the C=O stretching vibration. We then determined the resonance wavenumber in this model fibril as 1676 cm^−1^. For comparison, we also performed equilibrium MD simulations of an Aβ peptide for α-helix and random coil structures and calculated the infrared absorption spectra of these structures.

After the resonance wavenumber was determined, we performed nonequilibrium MD simulations of the Aβ amyloid fibril, applying a time-varying electric field with the resonance wavenumber to simulate the IR-FEL irradiation. To mimic the IR-FEL irradiation, an electric field was applied as a series of Gaussian-distributed pulses [82] with an interval of 35 ps. Each pulse is expressed as

(2) $$E(t)=E_{0}\exp\left(-\frac{{\left(t-t_{0}\right)}^{2}}{2\sigma^{2}}\right)\cos\left(\omega {\left(t-t_{0}\right)}^{2}\right)$$

where *E*_0_ is the maximum intensity of the electric field, *t* is time, *t*_0_ is the time at *E* = *E*_0_, σ is the standard deviation of the Gaussian distribution, and *ω* is the angular frequency related to the wavenumber *ν* such that *ω* = 2*πcν*, where *c* is the speed of light. The wavenumber *ν* was set to the resonance wavenumber 1676 cm^−1^, and *E*_0_ was set to 1 × 10^8^ V/cm. The value of σ was set to 1 ps to match that used in the IR-FEL experiments [185]. The final conformations and velocities in the previous equilibrium MD simulations were used as the initial conformations and velocities for the nonequilibrium MD simulations. Constant-temperature MD simulations were then performed at 310 K for 1000 pulses, that is, for 35 ns. Other simulation details can be found in Ref. [84].

### 7.2. Amyloid Fibril Disruption by Laser Irradiation

**Figure 15:**
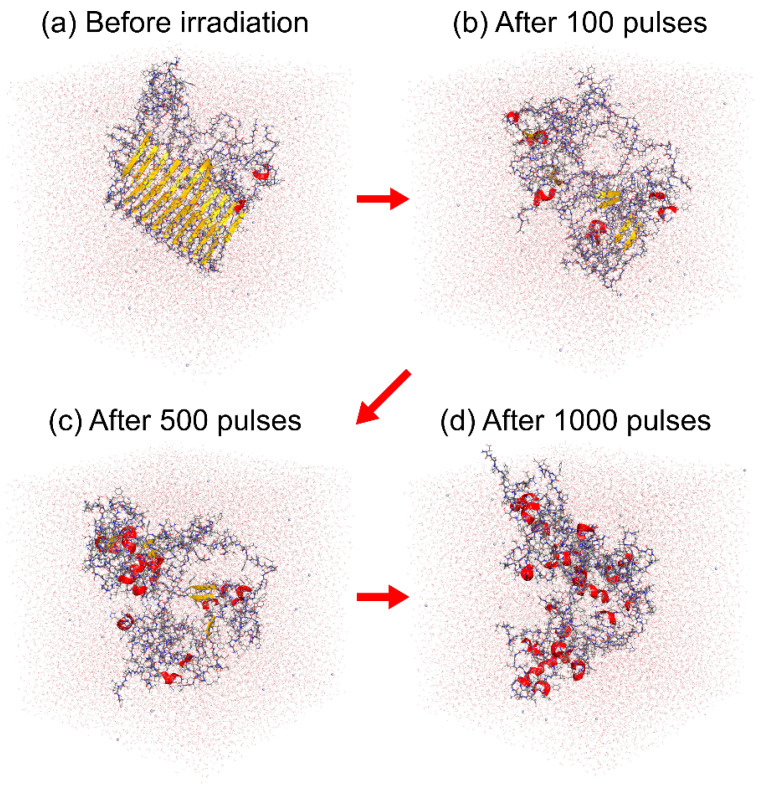
Snapshots during the laser-induced disruption process of the Aβ amyloid fibril in the nonequilibrium MD simulation (**a**) before IR-FEL irradiation, (**b**) after 100 pulses, (**c**) after 500 pulses, and (**d**) after 1000 pulses. The images were created using PyMOL [171].

**Figure 16:**
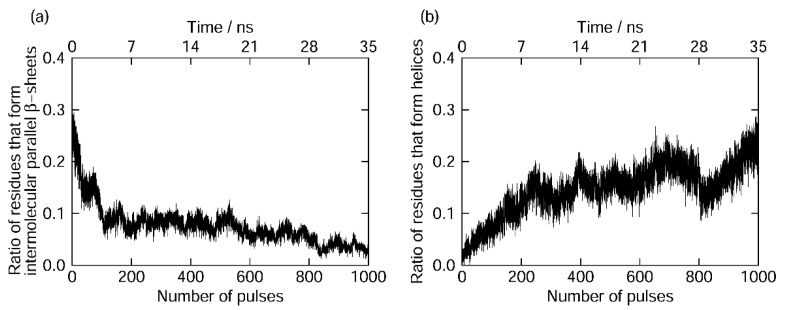
Time series of the ratio of the residues that form (**a**) intermolecular β-sheets and (**b**) helices in one of the nonequilibrium MD simulations.

We observed that the amyloid fibril was gradually destroyed, as shown in Figure 15. To quantify this result, we calculated the ratio of the amino acid residues that formed the intermolecular parallel β-sheet structure according to the DSSP criteria [190], as shown in Figure 16a. Almost all the intermolecular β-sheet structures were destroyed after 1000 pulses. In Figure 15, we see that many helix structures (red ribbons) formed after the amyloid fibril was disrupted. We calculated the ratio of the amino acid residues in the helix structures, as shown in Figure 16b. Here, the α-, 3_10_-, and π-helices [190] were included in the helix structures. This figure shows that the helix structures increased as the intermolecular β-sheet structure was destroyed in the MD simulations. These results are consistent with the IR-FEL experiments [185].

**Figure 17:**
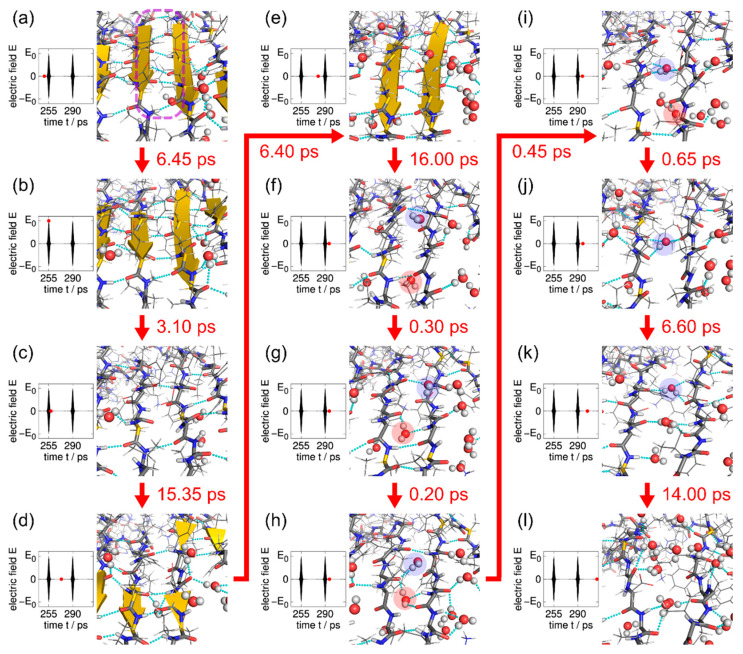
(**a**–**l**) Disruption process of the hydrogen bonds between the Aβ peptides and the electric field pulse. Two water molecules that disrupted the hydrogen bond re-formation between the Aβ peptides are highlighted with pink and blue circles. The images were created using PyMOL [171]. Reprinted with permission from Ref. [84]. Copyright 2021 American Chemical Society.

To examine the role of water molecules in the amyloid fibril disruption, we focused on the intermolecular β-bridges between two Aβ peptides. Figure 17 shows enlarged snapshots of the Aβ amyloid fibril in a typical MD simulation run and the electric field pulse intensity at the same time (red circles). Six intermolecular hydrogen bonds existed between the two β-strands (inside the purple dashed line) in Figure 17a. These intermolecular hydrogen bonds were broken by an electric field pulse in Figure 17b, and most of them were broken by the end of the pulse (Figure 17c). However, these hydrogen bonds re-formed after the pulse. During this re-formation, water molecules sometimes formed hydrogen bonds with the Aβ peptides (Figure 17d), but they soon separated from the peptides. The two β-strands were eventually completely repaired (Figure 17e). Before this pulse, the intermolecular hydrogen bonds between the Aβ peptides were repeatedly broken and repaired after each electric field irradiation in the same way. Immediately after the hydrogen bonds between the Aβ peptides were broken by the next pulse (Figure 17f), however, a water molecule (the pink-highlighted water molecule) entered the space between C=O and N–H, where the intermolecular hydrogen bond had been previously formed (Figure 17g). This water molecule formed hydrogen bonds with the Aβ peptides and prevented the hydrogen bond re-formation between C=O and N–H of the Aβ peptides in Figure 17h). Another water molecule (the blue-highlighted water molecule) also entered the space between the Aβ peptides and formed hydrogen bonds with the Aβ peptides. Some hydrogen bonds of the red-highlighted water molecule were broken in Figure 17i, but the blue-highlighted water molecule still formed some hydrogen bonds with the Aβ peptides. Even after the red-highlighted water molecule separated from the peptides, the blue-highlighted water molecule stayed in this location (Figure 17j). Other water molecules then entered the gap between the Aβ peptides (Figure 17k). Because the hydrogen bonds between the Aβ peptides were replaced by those between the Aβ peptides and the water molecules, the intermolecular hydrogen bonds between the Aβ peptides could not be re-formed before the next laser pulse. As a result, the intermolecular β-sheet of the Aβ amyloid fibril was destroyed (Figure 17l). This phenomenon occurred throughout the amyloid fibril, and the entire fibril was finally disrupted.

**Figure 18:**
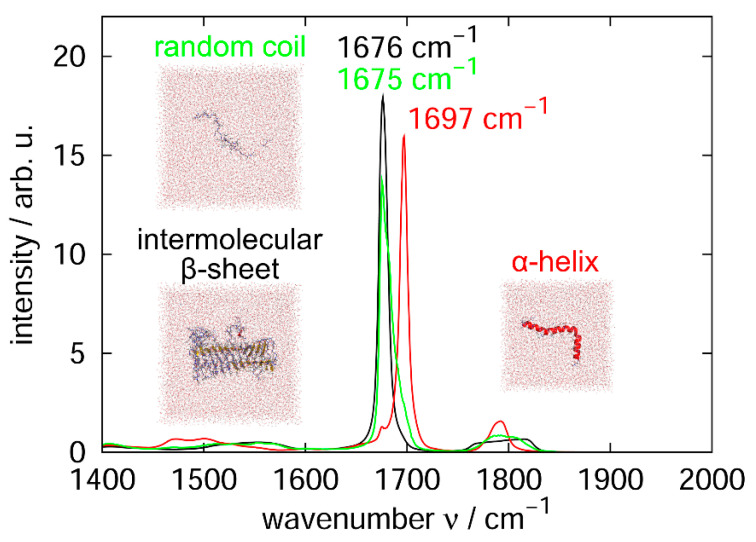
Infrared absorption spectra of the backbone C=O stretching vibration that forms the amyloid fibril (black), α-helix (red), and random coil (green). The snapshot images were created using PyMOL [171]. Reprinted in part with permission from Ref. [84]. Copyright 2021 American Chemical Society.

To understand why helix structures increased after the laser irradiation, we performed additional equilibrium MD simulations for α-helix and random coil structures of an Aβ peptide. We then calculated the infrared absorption spectrum of the C=O stretching vibration, as shown in Figure 18. We found that the resonance wavenumber for the random coil structure was 1675 cm^−1^, which is close to that for the intermolecular β-sheet structure and the laser wavenumber of this study, while the resonance wavenumber for the α-helix structure was 1697 cm^−1^, which is far from these wavenumbers. These results mean that helix structures can exist stably without breaking the hydrogen bonds between C=O and N–H because their resonance frequency is different from the laser frequency used to destroy the intermolecular β-sheet structure.

## 8. Conclusions

In this review, we presented the molecular dynamics (MD) simulation studies of full-length Aβ peptides and Aβ fragments that revealed the mechanism of their aggregation, the inhibition of the aggregation, the amyloid fibril in equilibrium, and the disruption of the amyloid fibril at the atomic level. We first explained that a β-hairpin structure enhances the formation of an intermolecular β-sheet structure. The β-hairpin structure is more formed at hydrophilic/hydrophobic interfaces. This is one of the reasons that the aggregation of the peptides is accelerated at the interfaces. The other reason is that the Aβ peptide has both hydrophilic and hydrophobic residues and tends to exist at the interfaces.

We also explained how polyphenols such as myricetin and rosmarinic acid interact with an Aβ(16–22) peptide. Because the aggregation of Aβ(16–22) peptides is caused by the electrostatic interaction between charged amino acid residues, Lys16 and Glu22, these polyphenols are expected to inhibit the aggregation by forming hydrogen bonds between these charged residues and the hydroxy and carboxyl groups of the polyphenols.

When almost all of the Aβ peptides in solution form amyloid fibrils, the system reaches equilibrium. The MD simulations of the Aβ amyloid fibril in equilibrium showed that Aβ always takes a closed form at the even end, whereas Aβ fluctuates more and takes an open form at the odd end. The reason for this phenomenon was also clarified. This finding is useful for understanding the mechanism of the amyloid fibril elongation and for designing drugs that inhibit its elongation.

It is possible to destroy the Aβ amyloid fibril by applying ultrasonic waves or infrared laser. The MD simulations also revealed the mechanisms of the Aβ amyloid fibril destruction for the ultrasonic wave and infrared laser irradiations. When the ultrasonic waves are applied, the Aβ amyloid fibril is disrupted by cavitation: a bubble is formed when the pressure is negative, and a water droplet then attacks and disrupts the amyloid fibril after the pressure becomes positive again. When the infrared laser is irradiated, hydrogen bonds between C=O and N−H are broken, but most of them re-form after the laser pulse. However, a water molecule nearby sometimes happens to enter the gap between C=O and N−H. It inhibits the re-formation of the hydrogen bonds, leading to the disruption of the amyloid fibril. In both cases of the ultrasonic wave and infrared laser irradiations, water molecules play an essential role in disrupting the amyloid fibril.

All simulation studies described here are based on the all-atom model with the explicit water solvent. There are several all-atom force fields, such as AMBER14SB [141], CHARMM36m [191], and GROMOS54A7 [192]. Some studies examined the structure of Aβ peptides using these force fields to find the optimal force field [193,194,195]. Because the all-atom force fields have been improved over the years, it is desirable to use the best force field available at the time. While MD simulation based on the all-atom models has the advantage of analyzing phenomena at the atomic level, it is computationally time-consuming. Since protein aggregation simulations are particularly computationally demanding, simulation studies using implicit solvent models, such as the GB/SA model [196,197,198], and coarse-grained models, such as the AWSEM [199], MARTINI [200,201], and UNRES force fields [202,203], are also being conducted [204,205]. The implicit solvent models used to be employed often [33,34,51,96] but now are not often used for all-atom simulations because it is known that the interaction between water and solutes plays an important role in the aggregation [206,207] and disaggregation of the amyloid fibrils [80,84]. Although these coarse-grained models do not provide atomic-level details, they can save much computation time. As more simplified models, lattice models have also been used to simulate protein aggregation [208,209,210,211]. The lattice models are primarily used to elucidate more general physical principles rather than to examine individual protein aggregates biologically. Depending on the purpose, these models would also continue to be used to study protein aggregation.

There is no established therapy to destroy amyloid fibrils at this time by irradiating the brain of Alzheimer’s patients with ultrasonic waves or infrared laser. However, we believe that there is a possibility that such a therapy can be realized in the future. In fact, animal experiments have been conducted to remove Aβ aggregates by irradiating the brain with ultrasonic waves, although its purpose is not to disrupt the Aβ aggregates [212,213,214,215]. Delivering therapeutic agents, such as anti-Aβ antibodies, to the brain is a possible approach for Alzheimer’s disease. However, the penetration of the therapeutic agents to the brain is hampered by the blood–brain barrier. To make it possible, focused ultrasound is utilized. Focused ultrasound opens the blood–brain barrier and promotes the therapeutic agent delivery to the brain. It was reported that the Aβ aggregates were reduced in Alzheimer’s disease model mice, and their behavior was improved [214].

We believe that the destruction of amyloid fibrils by the infrared laser irradiation may also have therapeutic potential in the future. In particular, it is noteworthy that the α-helix structure is formed more after the amyloid fibrils are destroyed by the infrared laser irradiation. This is because, unlike the β-hairpin structure, the α-helix structure can be maintained in the monomeric state and relatively easily excreted from the human body. Techniques to destroy amyloid fibrils may also be useful in developing treatments for other diseases caused by other amyloid fibrils.

As we reviewed here, MD simulation can identify which residues or atoms are important for the aggregation and aggregation inhibition and can be used to design a useful drug molecule for the treatment of Alzheimer’s disease and other neurodegenerative diseases. MD simulation can also elucidate the molecular mechanism of amyloid fibril destruction. We hope that MD simulation will become a new tool for developing treatments for these diseases in the future.

## Supplementary Materials

The following supporting information can be downloaded at: https://www.mdpi.com/article/10.3390/molecules27082483/s1, Figure S1: The chemical structures of (a) myricetin (Myr) and (b) rosmarinic acid (RA); Figure S2: Time series of the set pressure, which varies sinusoidally. References [31,80] are cited in the supplementary materials.
