# Supplementary material for: Molecular Dynamics Simulation Studies on the Aggregation of Amyloid-β Peptides and Their Disaggregation by Ultrasonic Wave and Infrared Laser Irradiation
Source: Molecules. 2022 Apr 12;27(8):2483. doi: 10.3390/molecules27082483 (PMC9030874; doi:10.3390/molecules27082483)
Supplement: Supplementary file 1 [file molecules-27-02483-s001.zip › molecules-1651802-supplementary.pdf]

# Supplementary Material

# Molecular Dynamics Simulation Studies on the Aggregation of Amyloid-β Peptides and Their Disaggregation by Ultrasonic Wave and Infrared Laser Irradiation

**Hisashi Okumura [1,2,3,*] and Satoru G. Itoh [1,2,3]**

[1] Exploratory Research Center on Life and Living Systems, National Institutes of Natural Sciences, Okazaki 444-8787, Aichi, Japan; itoh@ims.ac.jp

[2] Institute for Molecular Science, National Institutes of Natural Sciences, Okazaki 444-8787, Aichi, Japan

[3] Department of Structural Molecular Science, SOKENDAI (The Graduate University for Advanced Studies), Okazaki 444-8787, Aichi, Japan

* Correspondence: hokumura@ims.ac.jp

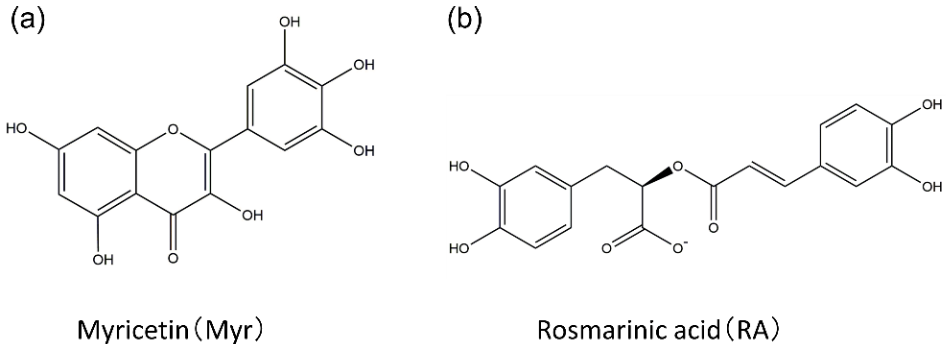


**Figure S1.** The chemical structures of (a) myricetin (Myr) and (b) rosmarinic acid (RA). Reprinted with permission from Ref. [1]. Copyright 2020 Elsevier.

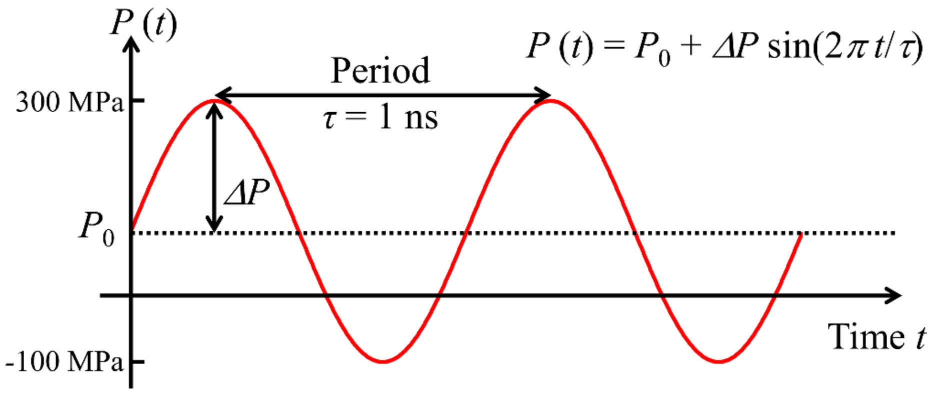


**Figure S2.** Time series of the set pressure, which varies sinusoidally. Reprinted with permission from Ref. [2]. Copyright 2014 American Chemical Society.

## References

1. Ngoc, L.L.N.; Itoh, S.G.; Sompornpisut, P.; Okumura, H. Replica-permutation molecular dynamics simulations of an amyloid-β(16–22) peptide and polyphenols. *Chem. Phys. Lett.* **2020**, *758*, 137913.
2. Okumura, H.; Itoh, S.G. Amyloid Fibril Disruption by Ultrasonic Cavitation: Nonequilibrium Molecular Dynamics Simulations. *J. Am. Chem. Soc.* **2014**, *136*, 10549–10552.
